# Vapor-Driven Propulsion of Catalytic Micromotors

**DOI:** 10.1038/srep13226

**Published:** 2015-08-18

**Authors:** Renfeng Dong, Jinxing Li, Isaac Rozen, Barath Ezhilan, Tailin Xu, Caleb Christianson, Wei Gao, David Saintillan, Biye Ren, Joseph Wang

**Affiliations:** 1 University of California, San Diego, Nanoengineering, La Jolla, 92093, United States; 2 South China University of Technology, Research Institute of Materials Science, Guangzhou, 510640, China; 3 University of California, San Diego, Department of Mechanical and Aerospace Engineering, La Jolla, 92093, United States

## Abstract

Chemically-powered micromotors offer exciting opportunities in diverse fields, including therapeutic delivery, environmental remediation, and nanoscale manufacturing. However, these nanovehicles require direct addition of high concentration of chemical fuel to the motor solution for their propulsion. We report the efficient vapor-powered propulsion of catalytic micromotors without direct addition of fuel to the micromotor solution. Diffusion of hydrazine vapor from the surrounding atmosphere into the sample solution is instead used to trigger rapid movement of iridium-gold Janus microsphere motors. Such operation creates a new type of remotely-triggered and powered catalytic micro/nanomotors that are responsive to their surrounding environment. This new propulsion mechanism is accompanied by unique phenomena, such as the distinct off-on response to the presence of fuel in the surrounding atmosphere, and spatio-temporal dependence of the motor speed borne out of the concentration gradient evolution within the motor solution. The relationship between the motor speed and the variables affecting the fuel concentration distribution is examined using a theoretical model for hydrazine transport, which is in turn used to explain the observed phenomena. The vapor-powered catalytic micro/nanomotors offer new opportunities in gas sensing, threat detection, and environmental monitoring, and open the door for a new class of environmentally-triggered micromotors.

The development of micro/nanoscale synthetic motors that convert energy into movement has been a fascinating research area of considerable fundamental and practical interest^1^,^2^,^3^,^4^,^5^,^6^,^7^,^8^,^9^,^10^. A variety of artificial micro/nanomotors propelled by chemical reactions or external stimuli has been developed over the past decade to overcome the challenges of propulsion at low Reynolds numbers and the effects of Brownian motion^11^. Particular attention has been given to chemically-powered catalytic motors, including bimetal nanowires^12^,^13^,^14^,^15^,^16^,^17^, spherical Janus micromotors^18^,^19^,^20^,^21^,^22^, and tubular microengines^6^,^7^,^23^,^24^,^25^,^26^,^27^,^28^, that exhibit autonomous self-propulsion in the presence of hydrogen peroxide fuel. Unfortunately, the requirement of the hydrogen peroxide fuel greatly impedes many practical applications of such catalytically propelled micro/nanomotors, and has led to the exploration of alternative chemical fuels. Diatomic halogens have thus been proposed to power bisegment Cu-Pt nanowires^29^, while acidic and chloride-rich water solutions have been used to power Zn microengines and Mg Janus micromotors^30^,^31^,^32^,^33^, respectively. Gao *et al.* have shown recently that ppb–ppm levels of hydrazine can also be used as an extremely efficient fuel to power iridium-based Janus micromotors^34^. Such hydrazine levels have been shown to exert negligible toxicity on different animals^35^,^36^. However, a common drawback of all chemically-powered micro/nanomotors is that they must operate in solutions prepared with such additional fuel materials. There are no previous reports about using the atmospheric environment to trigger the motion of micro/nanomotors.

This paper demonstrates the first example of catalytic micromotors powered by vapor-phase chemicals present in their surrounding atmosphere. The new concept of micromotors harvesting the chemical fuel from their own surrounding atmospheric environment is illustrated using catalytic iridium-gold (Ir-Au) Janus microsphere motors in the presence of hydrazine vapor. The self-electrophoretic movement of Ir-Au micromotors in the presence of hydrazine fuel has been shown to propel these microsphere motors even at very low (nanomolar to micromolar) fuel concentrations^34^. In the present work, we use this behavior to demonstrate the first example of remotely triggered high-speed micromotors. Partition of hydrazine from the surrounding atmosphere into the micromotor solution is shown to trigger the efficient movement of Ir-Au microsphere motors without direct addition of the hydrazine fuel to the sample solution. The resulting vapor-driven catalytic micromotors demonstrate distinct ‘Off-On’ response to fuel molecules in the surrounding atmosphere and move at remarkable speeds of 30 body lengths per second. This operation obviates the need for mixing together the fuel and micromotor solutions, and creates a new type of remotely-triggered and powered catalytic micromotors that are responsive to volatile species in their surrounding environment. Such ability of catalytic micromotors to swim in response to changes in surrounding atmosphere or by sensing a remote chemical source holds considerable promise for diverse environmental and defense applications.

## Results

**Figure 1:**
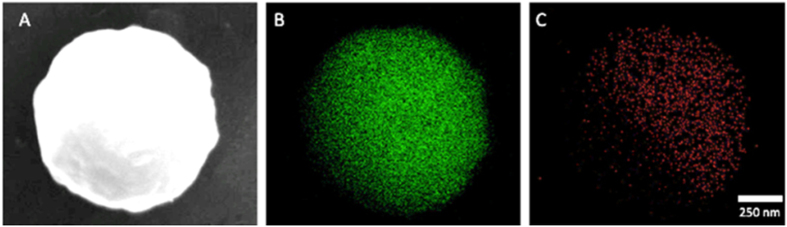
(**A**) Scanning electron microscopy (SEM) image of the Au/Ir motor. (**B**,**C**) Energy-dispersive X-ray (EDX) spectroscopy images illustrating the distribution of the gold inner core and iridium catalytic patch, respectively.

The Janus micromotors consist of gold particles (1.15 *μ*m diameter) with one hemisphere coated with iridium metal. Iridium metal catalyst beds are commonly utilized to decompose hydrazine fuel. Such Janus motors are easily fabricated by a directional Ir sputter deposition onto a monolayer of gold microparticles dispersed onto a glass slide. The scanning electron microscopy (SEM) image of the fabricated Janus motor and corresponding energy-dispersive X-ray (EDX) characterization are displayed in Fig. 1.

**Figure 2:**
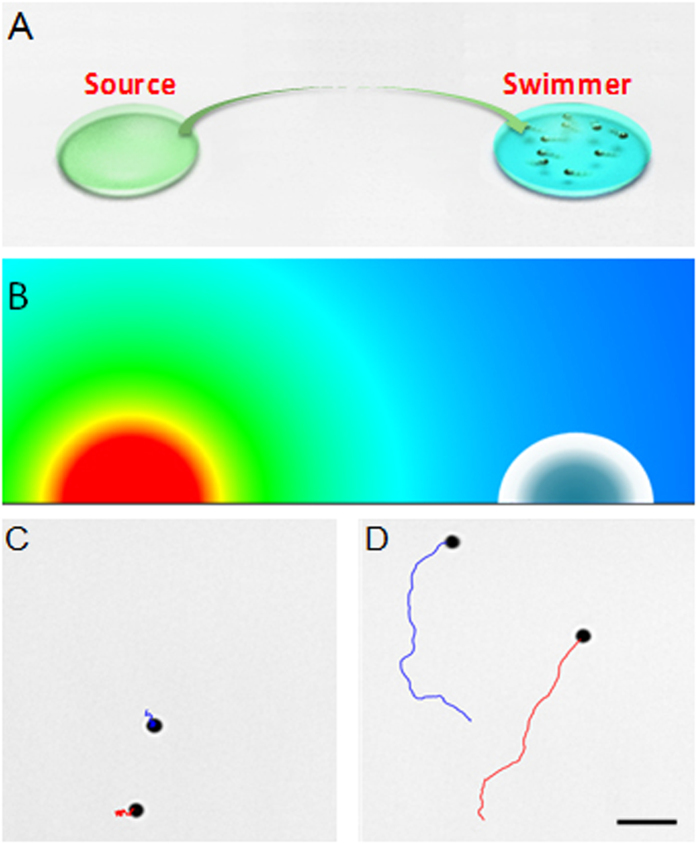
Catalytic micromotors powered by a remote fuel source. (**A**) Propulsion of Ir-Au micromotors by external hydrazine diffusing from the fuel source droplet through the surrounding atmosphere into the motor droplet. (**B**) Simulation of the hydrazine concentration gradient produced by the hydrazine source (left) and the hydrazine concentration gradient generated within the sample droplet (right) based on a theoretical model for hydrazine transport. (**C**,**D**) Track lines of the motion of Ir-Au motors over 2 seconds, taken from SI Video 2, before (**C**) and 15 seconds after (**D**) placing the 20% hydrazine droplet (1 *μ*L; diameter: 2.5 mm) 0.5 cm away from the micromotor droplet (1 *μ*L; diameter: 2.5 mm). Scale bar, 10 *μ*m.

To demonstrate the vapor-powered propulsion of Ir-Au micromotors, we rely on the volatilization of hydrazine from a nearby source and its diffusion through the surrounding atmosphere into the motor droplet (Fig. 2A). The self-propulsion of such Ir-Au Janus micromotors is efficient enough that they can be triggered by hydrazine molecules diffusing through the atmosphere from a remote hydrazine source (Fig. 2A). Such large-scale micromotor motion, remotely triggered by a hydrazine droplet, placed at a 1 cm distance, is demonstrated in SI Video 1. In our experiments, the two droplets - containing the fuel and motors - were placed nearby each other on a glass slide within a containment enclosure atop the microscope stage, in order to minimize the potential effects of atmospheric convective streams. The rapid diffusion of hydrazine from the surface of the fuel source droplet leads to the formation of a vapor-phase hydrazine concentration field in the surrounding air. The dissolution of vapor-phase hydrazine into the micromotor droplet and subsequent internal diffusion create a gradient of the hydrazine fuel within the motor droplet. Such gradients in the surrounding air and within the motor droplet are illustrated in the simulated profiles, shown in Fig. 2B, based on a theoretical model for hydrazine transport (discussed in detail in Supporting Information). The resulting gradient within the motor droplet leads to distinct position-concentration dependent speed variations.

Figure 2(C,D) displays tracking lines, taken from SI Video 2, illustrating the Brownian movement of the Ir-Au micromotors in the fuel-free sample droplet prior to the placement of the hydrazine fuel source (Fig. 2C), and their acceleration to an average speed of 10 *μ*m/s 15 seconds after placing the hydrazine droplet (concentration 20%) at a separation distance of 0.5 cm (Fig. 2D). Unlike previous experiments involving direct fuel mixing, where micromotors immediately and uniformly reach their maximum speed upon adding the fuel, the present vapor-based propulsion mechanism leads to the formation of concentration gradients within the micromotor droplet (simulated below), and hence to several new phenomena such as a distinct spatio-temporal speed dependence. These include gradual acceleration of the micromotors as well as spatial speed variation for motors located at different positions within the droplet. In addition, the new vapor propulsion mechanism allows control of the micromotor speed by changing the source-sample separation distance or the hydrazine concentration in the source.

The rate of hydrazine vapor diffusion into the micromotor sample is a function of both the hydrazine-source concentration and the source-sample separation distance. Evaporation of hydrazine from the source droplet creates a concentration *c*_*s*_ of vapor phase hydrazine on the surface of the fuel (source) droplet. *c*_*s*_ is directly proportional to the level of hydrazine inside the source droplet. Because of the high diffusivity of hydrazine in air, its vapor concentration quickly reaches steady state in the vicinity of the source. The steady state vapor phase hydrazine concentration immediately surrounding the sample droplet *c*^*s*^_*a*_(*L*) is solved as


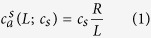


where *R* is the source droplet radius and *L* is the separation distance between the two droplets containing the fuel and micromotor solution (see supporting information)^37^.

**Figure 3:**
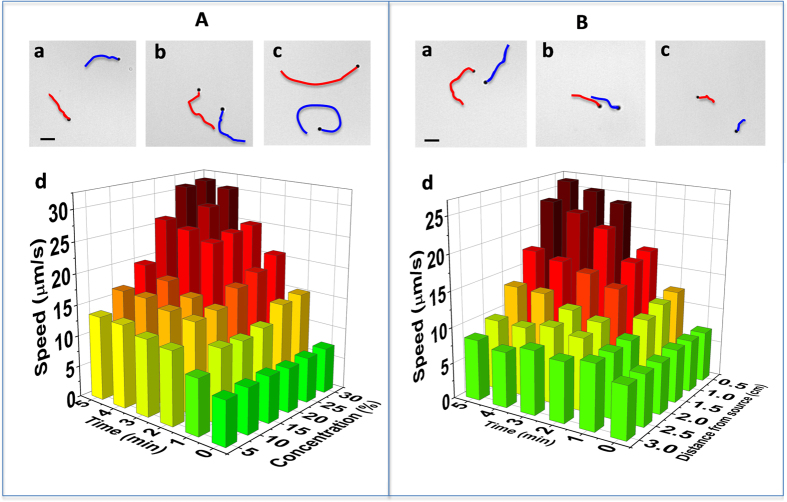
Spatio-temporal speed dependence of vapor-powered micromotors. (**A**) Dependence of the motor speed upon time and fuel concentration at a fixed separation distance of 0.5 cm. (a–c) Track lines of the micromotor motion, over a 2 sec period, taken from SI Video 3, 5 min after placing droplets of 10%, 20%, and 30% hydrazine, respectively, 0.5 cm apart. (**B**) Dependence of the motor speed upon the time and separation distance using a source droplet containing 20% hydrazine. (a–c) Track lines of the micromotor motion, over 2 second periods, taken from SI Video 4, 5 min after placing the fuel droplet containing 20% hydrazine at separation distances of 1, 2, and 3 cm, respectively. (d) Corresponding 3D plots. Scale bar, 10 μm. Droplet diameter and volume: 2.5 mm and 1 μL, respectively.

A lower source concentration leads to a lower hydrazine vapor concentration in the vicinity of the motor droplet *c*^*s*^_*a*_(*L*) due to their linear relationship shown in equation (1). Our experiments demonstrated that a decrease in the hydrazine source concentration indeed leads to a decrease in the average motor speed over time (Fig. 3A), although the speeds are still markedly higher than those due to Brownian fluctuations over the entire 5 minutes period. The dependence of the micromotor speed on the hydrazine fuel concentration was examined by directly adding the fuel solution to the sample (supporting Figure S1). The significantly faster motor speeds due to higher hydrazine concentrations are illustrated in the track lines of the motor movement Fig. 3A(a–c) and the corresponding SI Video 3. Ordinarily, when the Ir-Au micromotors are directly mixed with high concentrations of the hydrazine fuel (≥0.001% level), a strong inhibiting effect arises due to the high concentration of the ionic conjugate species of both hydrazine and water (N_2_H_5_^+^ and OH^−^, respectively)^34^. In the case of vapor propulsion, however, no deceleration due to this inhibiting ionic-strength effect has been observed. Our results indicate that the local hydrazine concentration within the motor sample due to vapor dissolution lies solely below the 0.001% level, even upon exposure to a high source concentration of 30% at close proximity to the micromotor droplet.

The source-sample separation distance has a profound effect upon the micromotor speed, as well as on the acceleration behavior of the micromotors. The average motor speed decreases significantly upon increasing the separation distance *L* between fuel source and motor droplet (Fig. 3B), reflecting the lower steady-state concentration of hydrazine over larger distances due to their inverse linear relationship (eq. 1). At short separation distances, the Janus micromotors are able to continuously accelerate over a five-minute period to maximum speeds of 26 *μ*m/s. This acceleration is greatly diminished using larger separation distances. For example, at the largest separation distance the motors were found to accelerate to their peak speeds of 7 *μ*m/s only after 30 seconds. The effect of different separation distances on the motor speed is illustrated from the 1 second motion track lines of Fig. 3B(a–c), following a five minute exposure to the fuel droplet. At all separation distances examined, the motors still moved faster than due to Brownian motion alone, highlighting the sensitivity of these micromotors to ultralow levels of hydrazine.

**Figure 4:**
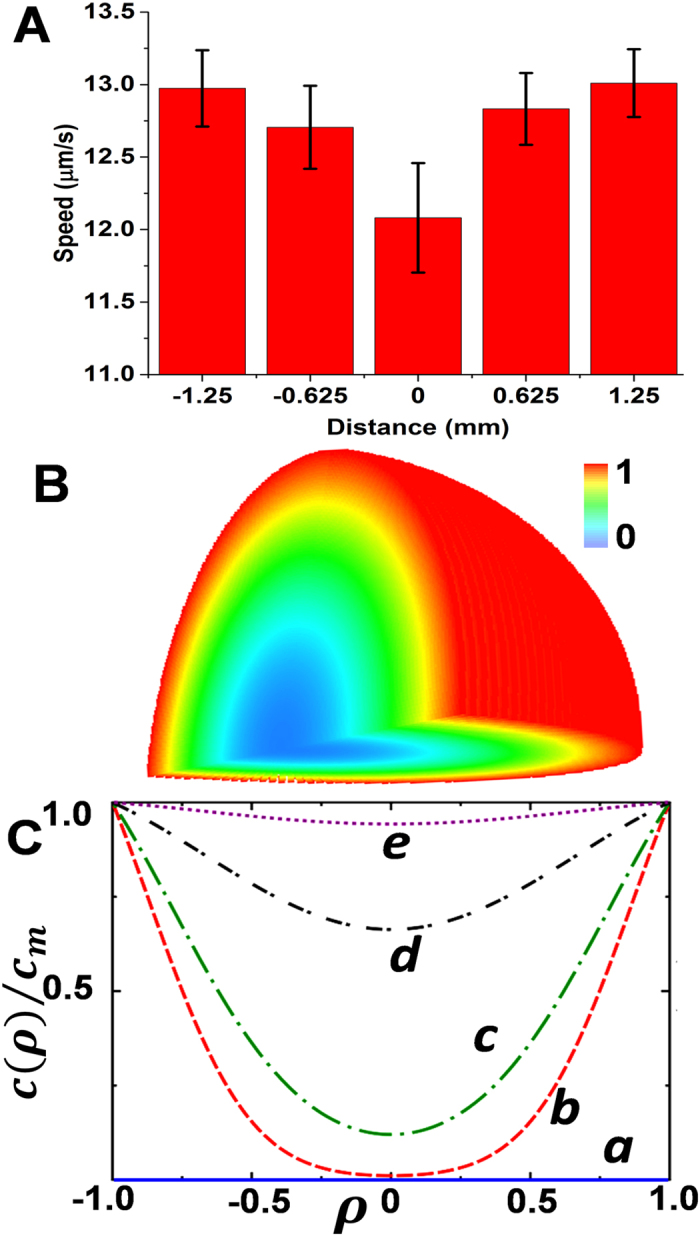
Profiles of the hydrazine level and the motor speed within the motor droplet. (**A**) Motor speeds at several radial distances from the center within the sample droplet after a one min exposure to the 20% hydrazine source droplet (the negative distance being closest to the source, and positive being furthest). Droplets (2.5 mm diameter, 1 *μ*L) separated by 0.5 cm. (**B**) 3D simulated plot of the normalized hydrazine concentration within the sample droplet 1 min after exposure. (**C**) Normalized hydrazine concentration profile as a function of the radial position (*ρ* = *r/R*) within the sample droplet for different times after placing the hydrazine source: 0, 0.5, 1, 2.5, and 5 min (a–e).

An interesting phenomenon that arises due to the diffusion involved in the vapor propulsion mechanism is that for a short time after exposure to the fuel droplet, the motor speed depends upon the location of the motor within the sample droplet itself, and specifically on its distance from the free surface of the droplet. Although the environmental hydrazine immediately surrounding the motor droplet reaches a steady-state concentration within approximately two seconds after the start of diffusion, it takes approximately 15 minutes to reach a uniform concentration within the 2.5 mm diameter water droplet containing the micromotors, owing to its 20,000-fold lower diffusivity in water. This leads to a significant variation in the fuel concentration within the motor droplet at short times. This is consistent with the results from our mathematical model (see SI) where the transport of hydrazine within the motor droplet is treated as a diffusion process within the hemisphere. We assume radial symmetry, which inherently captures the no-flux of hydrazine through the flat substrate, thus allowing us to solve the problem in spherical coordinates. The simulated hydrazine concentration profiles within the motor droplet, shown in Fig. 4(B,C) and SI Video 5, illustrate such decrease in the local hydrazine concentration from the droplet edges to the center. As time increases, the hydrazine concentration increases within the domain and asymptotically reaches the boundary concentration *c*_*m*_. Accordingly, motors closer to the motor-droplet boundary (*ρ* = *r/R* close to 1) are expected to travel faster than those at the center. Overall, the data of Fig. 4A clearly illustrate that the partition of the fuel into the motor droplet, leads to distinct position-concentration dependent speed variations. In agreement with the theoretical predictions, we observed noticeably faster average motor speeds around the droplet edges compared to the center of the droplet (Fig. 4A). In addition, we found that motors at equivalent radial distances moved at similar speeds over the range of experimental separation distances considered here. These experimental data are in good agreement with our model, where the hydrazine concentration immediately surrounding the small 2.5 mm droplet is assumed to be uniform on the scale of the droplet. This also serves as confirmation of the minimal effects of potentially disruptive convective streams outside the sample droplet, as such streams would disturb the surrounding hydrazine concentration and contribute to different speeds at similar radial distances, counter to our experimental observations. Furthermore, the significant spatial dependence of the micromotor speed indicates the negligible effects of convective streams within the sample droplet, such as those due to evaporation. If such flows were present and strong, this would lead to mixing and enhancement of the hydrazine transport inside the motor droplet, which could be modeled as an effective diffusivity (at long times). The timescale for hydrazine transport within the motor droplet - calculated from pure diffusion - matches our experiments, however indicating that the potential effect of convective flow-induced transport in our study is at best weak. Overall, the agreement of the observed dependence of the motor speed on the source-sample separation distance, hydrazine source concentration, and radial position within the motor droplet with the theoretical predictions validates our modeling approach and theoretical assumptions

In conclusion, we have demonstrated a new type of catalytic micromotors that are responsive to their surrounding environment. The movement of these motors is triggered remotely by a fuel vapor partitioned from the surrounding atmosphere into the micromotor solution. This new concept was illustrated using vapor-propelled iridium-based micromotors that display efficient motion of over 30 *μ*m/s in the presence of hydrazine vapor without adding the hydrazine fuel directly to the sample solution. Factors affecting such vapor-powered micromotor motion have been investigated using a theoretical model for hydrazine transport. Such vapor-powered micromotors have the capability to act as highly selective sensors for toxic gases, with a distinct ‘Off-On’ response to low atmospheric concentrations of the target vapor. Similarly, ‘On-Off’ gas sensing schemes may be developed by pollutants inhibiting the catalytic activity of the motors, analogous to the water toxicity testing based on the inhibition of catalase-based micromotors by solubilized toxins^38^. Pollutants affecting the motor speed may also be explored, analogous to the strong effect of trace silver ions upon the propulsion of Au-Pt nanowire motors, as well as for the enhanced detection of Ag-nanoparticle tagged DNA^39^,^40^,^41^. These motion-based sensing studies indicate the strong potential of the new vapor-powered motors to selectively detect airborne toxins. Future efforts will aim at exploiting new volatile fuels and new catalytic motors for expanding the concept of remotely-triggered movement towards the detection of different air pollutants. Such ability of micromotors to swim in response to the presence of a remote fuel source offers considerable promise for designing future environmentally-responsive micromachines for a wide range of important future defense and environmental applications.

## Methods

Synthesis of Janus micromotors. The Janus micromotors were prepared using gold microparticles (1.15 *μ*m mean diameter, Alfa Aesar, Ward Hill, MA, USA) as the base particles. 10 *μ*g of gold particles were first dispersed into isopropyl alcohol (A451-4, Fisher, Pittsburgh, PA, USA) and centrifuged. Then, the gold particles were redispersed in 150 *μ*L isopropyl alcohol. The sample was then spread onto glass slides and dried uniformly to form particle monolayers. The particles were sputter coated with a thin Ir layer using an Emitech K575X Sputter Coater for 3 cycles with 10 s per cycle. The Ir layer thickness was found to be 20 nm, as measured by the Veeco DEKTAK 150 Profilometer. The micromotors were subsequently released from the glass slides via pipette pumping and dispersed into double distilled water.

Speed calibration experiments. To determine the relationship between the Ir-Au motor speed and fuel concentration, aqueous hydrazine solutions (Sigma #309400) ranging from 0.0000002% to 20% were prepared and directly mixed with the motor droplets. The propulsion calibration experiments were performed by mixing 1 *μ*L of the motor and hydrazine solutions each.

Vapor experiments. Aqueous hydrazine solutions, ranging from 5% to 30%, were prepared for the vapor experiments. A 1 *μ*L droplet containing the micromotors was placed first on the glass slide within a containment enclosure atop the microscope stage, in order to minimize the potential effects of atmospheric convective streams. The diameter of the motor droplet was found to be 2.5 mm. After the motors settled into focus of the microscope, a 1 *μ*L droplet of hydrazine fuel was placed on the slide at a fixed separation distance from the motor droplet.

Equipment. Videos were captured by an inverted optical microscope (Nikon Instrument Inc. Ti-S/L100), coupled with 40x objectives, and a Hamamatsu digital camera C11440 using the NIS-Elements AR 3.2 software.

## Additional Information

**How to cite this article**: Dong, R. *et al.* Vapor-Driven Propulsion of Catalytic Micromotors. *Sci. Rep.* **5**, 13226; doi: 10.1038/srep13226 (2015).

## Supplementary Material

Supplementary Information

Supplementary Video S1

Supplementary Video S2

Supplementary Video S3

Supplementary Video S4

Supplementary Video S5
