# Supplementary material for: Vapor-Driven Propulsion of Catalytic Micromotors
Source: Sci Rep. 2015 Aug 18;5:13226. doi: 10.1038/srep13226 (PMC4540091; doi:10.1038/srep13226)
Supplement: Supplementary Information [file srep13226-s1.pdf]

Vapor-Driven Propulsion of Catalytic Micromotors

Renfeng Dong[a,b†], Jinxing Li[a†], Isaac Rozen[a], Barath Ezhilan[c], Tailin Xu[a], Caleb Christianson[a], Wei Gao[a], David Saintillan[c], Biye Ren[b*], Joseph Wang[a*]

[a]Department of Nanoengineering, University of California, San Diego, La Jolla, California 92093, United States.

[b]Research Institute of Materials Science, South China University of Technology, Guangzhou 510640, P. R. China

[c]Department of Mechanical and Aerospace Engineering, University of California, San Diego, La Jolla, California 92093, United States.

*Corresponding Author: josephwang@ucsd.edu, mcbyren@scut.edu.cn, dstn@ucsd.edu

[†]R.D. and J.L. contributed equally to this paper

Supporting Videos

SI Video 1. Large scale micromotor motion remotely triggered by placing a hydrazine fuel droplet (concentration 30%) at a distance of 1.0 cm from the micromotor droplet.

SI Video 2. Behavior of two micromotors before and after the remote placement of a hydrazine droplet (concentration 20%) 0.5 cm away from the micromotor droplet. .

SI Video 3. Micromotor motion 5 min after placing droplets containing 10%, 20%, and 30% hydrazine at a distance of 0.5 cm over 2 second periods.

SI Video 4. Micromotor motion 5 min after placing droplets of 20% hydrazine at different distances from the micromotor droplet: 1.0 cm, 2.0 cm, and 3.0cm over 2 second periods.

SI Video 5. 3D simulations of the normalized hydrazine concentration within the sample droplet for the first 4 minutes after exposure to the hydrazine fuel droplet. The videos are 6 times faster than real time.

Supporting Figure

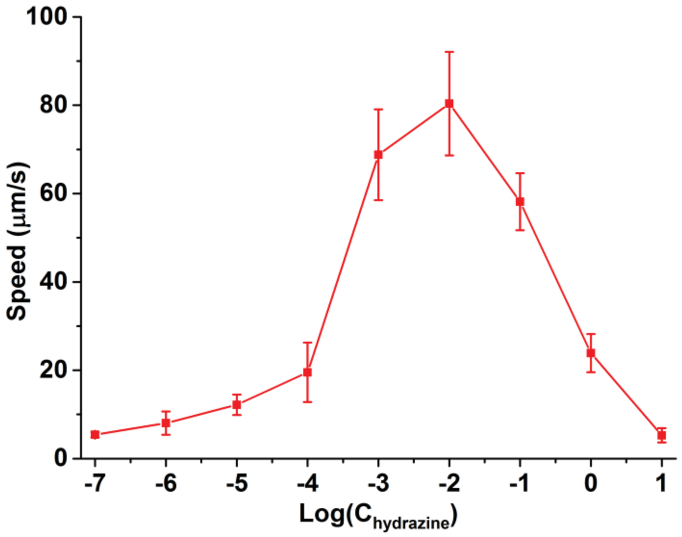

Figure S1*Dependence of the speed of Ir-Au Janus micromotors upon the hydrazine concentrations over the 0.0000001−10% range.*

Diffusion model and simulation.

The dynamics of the new system can be described using the following mathematical model. We assume the source and motor droplets to be hemispherical in shape. Let R be the radius of the droplets and L be the source-sample separation distance. Evaporation of hydrazine from the source droplet creates a vapor-phase hydrazine concentration c_s at the surface of the droplet. The source droplet surface concentration is directly proportional to the percentage of hydrazine inside the source droplet.

Vapor phase hydrazine diffuses from the surface of the source droplet driving the formation of concentration gradients in air. Hydrazine transport in air is modeled using the diffusion equation in spherical coordinates.

$$\frac{\partial c_a}{\partial t} = \frac{D_{Ha}}{r^2}\frac{\partial}{\partial r}\left(r^2\frac{\partial c_a}{\partial r}\right) \qquad (1)$$

subject to boundary conditions,

$$c_a(r = R, t > 0) = c_s \qquad (2)$$

$$c_a(r \to \infty, t \geq 0) = 0 \qquad (3)$$

and initial condition,

$$c_a(R < r < \infty, t = 0) = 0 \qquad (4)$$

where r is the radial distance from the center from the source droplet and D_{Ha} = 0.4164 cm^2/s is the diffusion coefficient of hydrazine in air. This equation can be solved analytically to obtain the vapor phase hydrazine concentration in the atmosphere: [1]

$$c_a(r,t) = c_s \frac{R}{r} \mathrm{Erfc}\left(\frac{r-R}{2\sqrt{D_{Ha}t}}\right). \qquad (5)$$

At long times (t >> 2.4s), the concentration of vapor-phase hydrazine in air reaches a steady state given by:

$$c_a^s(r) = c_s \frac{R}{r}. \qquad (6)$$

When the distance of separation between the droplets is larger than the radius of the droplet (L>>R), the vapor phase hydrazine concentration near the surface of the motor droplet can be considered to be uniform to leading order. While hydrazine diffuses in air with a timescale L^2/D_{Ha} = 2.4s, it diffuses inside the water droplet with a much slower timescale of R^2/D_{Hw} = 822s (where D_{Hw} =1.9e-005 cm^2/s is the diffusivity of hydrazine in water). Because of the separation of timescales, the vapor phase hydrazine concentration immediately surrounding the motor droplet can be approximated by the steady state concentration at the center of the motor droplet $c_a(L)$ given by:

$$c_a^s(L) = c_s \frac{R}{L}. \qquad (7)$$

This equation suggests that the vapor-phase hydrazine concentration surrounding the motor droplet has a linear relationship with the surface concentration of the source droplet (c_s) (and hence with the hydrazine concentration inside the source droplet) and an inverse linear relationship with the source-sample droplet separation distance (L). Therefore, the average speed within the motor droplet follows a similar relationship with these variables.

The dissolution of vapor-phase hydrazine into the motor droplet and the subsequent transport are modeled as a diffusion problem within a hemisphere according to the equation

$$\frac{\partial c_w}{\partial t} = \frac{D_{Hw}}{r^2}\frac{\partial}{\partial r}\left(r^2\frac{\partial c_w}{\partial r}\right) \tag{8}$$

subject to boundary and initial conditions

$$c_w(r = R) = c_m \tag{9}$$

$$c_w\left(0 < r < R, t = 0\right) = 0 \tag{10}$$

where r now refers to the radial position within the motor droplet measured from its center. c_m is proportional to $c_a^s(L)$ and both are related by Henry's Law.

The solution to this equation can be obtained numerically using finite differences and is shown in Figure 4(B-C) of the main text.

Reference:

1. Haberman, R. *Applied Partial Differential Equations with Fourier Series and Boundary Value Problems (5th Edition);* AMC(2013).
